# Treatment of chronic insomnia in elderly adults with dual orexin receptor antagonists: a systematic review and meta-analysis

**DOI:** 10.1093/sleep/zsag135

**Published:** 2026-05-17

**Authors:** Eleonora Rollo, Annibale Antonioni, Lamberto Manzoli, Francesco Di Lorenzo, Maria Elena Flacco

**Affiliations:** Center for Neurodegenerative Diseases and the Aging Brain, University of Bari Aldo Moro at Pia Fondazione “Card. G. Panico”, Tricase 73039, Italy; Department of Neurosciences, Università Cattolica del Sacro Cuore, Rome 00168, Italy; Department of Neuroscience and Rehabilitation, University of Ferrara, Ferrara 44121, Italy; IIT@UniFe Center for Translational Neurophysiology of Speech and Communication, Istituto Italiano di Tecnologia, Ferrara 44121, Italy; Department of Medical and Surgical Sciences, University of Bologna, Bologna 40100, Italy; Department of Environmental and Prevention Sciences, University of Ferrara, Ferrara 44121, Italy; Neuropsychophysiology Lab, Santa Lucia Foundation IRCCS, Rome 00179, Italy; Department of Environmental and Prevention Sciences, University of Ferrara, Ferrara 44121, Italy

**Keywords:** almorexant, chronic insomnia, daridorexant, DORAs, dual orexin receptor antagonists, elderly, lemborexant, polysomnography, sleep, suvorexant

## Abstract

**Study Objectives:**

Chronic insomnia is common among older adults, and its management is particularly challenging due to changes in sleep architecture, multimorbidity, and increased vulnerability to adverse drug effects. Dual orexin receptor antagonists have emerged as a novel therapeutic class with promising efficacy and tolerability profiles. However, evidence specific to older adults remains limited. Here, we performed the first systematic review and meta-analysis specifically evaluating the efficacy and safety of dual orexin receptor antagonists in elderly patients with chronic insomnia (PROSPERO CRD42024565687).

**Methods:**

Randomized controlled trials were identified from MEDLINE, Scopus, and ClinicalTrials.gov. Objective polysomnographic outcomes, including latency to persistent sleep, total sleep time, and wake after sleep onset, as well as subjective sleep measures, were analyzed, along with safety outcomes.

**Results:**

Ten studies met the inclusion criteria for the systematic review, with five studies contributing to the meta-analysis. The quantitative synthesis comprised 2235 patients in the experimental group and 1409 patients in the control group. Overall, compared with placebo dual orexin receptor antagonists reduced wake after sleep onset and latency to persistent sleep while increasing total sleep time after 1 month and 3 months of treatment. Importantly, dual orexin receptor antagonists increased subjective total sleep time. Finally, daridorexant showed a favorable safety profile, whereas lemborexant and suvorexant were associated with higher rates of treatment-emergent adverse events than placebo, without an increase in serious adverse events.

**Conclusions:**

Despite limited evidence, dual orexin receptor antagonists appear effective and generally safe for treating chronic insomnia in the elderly, supporting their role as a valuable therapeutic option.

## Introduction

Chronic insomnia (CI) is a common sleep condition with an increased incidence in people older than 65 years [1]. Specifically, CI is characterized by difficulty initiating or maintaining sleep, or by early-morning awakening with an inability to resume sleep [2]. Importantly, to establish a clinical diagnosis, nocturnal disturbances must affect daytime functioning, causing excessive sleepiness, irritability, impairments in concentration and memory, social and work difficulties, and mood issues such as anxiety or depression, often accompanied by ongoing worries about sleep quality and its impact on daily life [3]. These issues are even more critical in older adults, who not only exhibit age-related changes in sleep architecture but also often have multiple comorbidities and complex medication regimens that further impair sleep quality [1, 4, 5]. This cluster of factors makes the management of insomnia particularly challenging in this population [6]. Notably, sleep disturbances in the elderly not only lead to a significant decline in quality of life but also raise the risk of falls and cognitive issues [7–9]. These effects can ultimately result in a progressive loss of autonomy and independence in daily activities, imposing substantial social, economic, and healthcare burdens. Therefore, identifying effective therapeutic approaches to manage CI in older adults is a key focus of ongoing research.

Although cognitive behavioral therapy is recommended as the primary treatment for CI, it is not always available in all healthcare settings [10, 11]. As a result, patients often have limited options and may turn to more easily accessible pharmacological agents, such as benzodiazepines (BDZ), benzodiazepine receptor agonists (BZRA), and antidepressants. However, their side effects may be problematic, particularly for older adults [6, 12]. Recently, dual orexin receptor antagonists (DORAs) have been approved as a new pharmaceutical class for the treatment of CI [13]. Numerous studies have shown that DORAs are effective in treating insomnia and have a favorable safety profile [14–18]. Improvements in key sleep parameters have been documented using polysomnography (PSG), with significant benefits in objective sleep measures, including sleep latency (SL), total sleep time (TST), and wake after sleep onset (WASO) [18]. Moreover, patients reported improved sleep quality and satisfaction, as measured by validated self-report questionnaires and other patient-reported outcome measures [17–19]. Together, these findings support the multidimensional effectiveness of DORAs in addressing both the physiological and experiential aspects of CI.

However, most studies on DORAs have involved young to middle-aged patients [18]. As a matter of fact, evidence on the effectiveness and safety of DORAs in older adults remains limited. To address these gaps, to the best of our knowledge, we conducted the first systematic review and meta-analysis of studies on the efficacy and safety of DORAs in elderly individuals. We believe this goal is timely and highly relevant, as it can inform clinical decisions and support the use of this promising therapeutic class in a particularly fragile and clinically complex patient group, where careful evaluation of the risk–benefit profile is essential.

## Materials and methods

### Search strategy

This systematic review and meta-analysis followed the updated version of the Preferred Reporting Items for Systematic Reviews and Meta-Analyses (PRISMA) guidelines (Supplementary file 1) [20], and the study protocol was registered in the “International Prospective Register of Systematic Reviews” (PROSPERO, CRD42024565687). MEDLINE (via PubMed), Scopus (via EMBASE), and ClinicalTrials.gov databases were searched through March 31, 2025, and were frequently updated through November 15, 2025, for studies reporting efficacy and safety data on DORAs for CI in the elderly population. Specifically, our PICO question was: P: elderly adults (≥65 years) with CI; I: treatment with DORAs; C: placebo or other hypnotics; O: efficacy and safety.

The primary efficacy outcomes were: (1) latency to persistent sleep (LPS); (2) WASO; (3) sleep efficiency (SE), and (4) TST, as determined by objective sleep assessment (i.e. PSG). As additional outcomes, we examined efficacy measures derived from subjective sleep parameters, including subjective TST (sTST), subjective SE (sSE), subjective SL (sSL), and subjective WASO (sWASO).

Regarding safety, we evaluated adverse events (AEs), specifically the incidence of TEAEs and serious AEs (SAEs).

The following search strategy was used, tailored according to the specific database: “insomnia”[All Fields] OR “insomnia”[MeSH Terms] OR “sleep initiation and maintenance disorders”[MeSH Terms] OR (“sleep”[All Fields] AND “initiation”[All Fields] AND “maintenance”[All Fields] AND “disorders”[All Fields]) OR “sleep initiation and maintenance disorders”[All Fields] OR “chronic insomnia”[All Fields]) AND (“aged”[MeSH Terms] OR “aged”[All Fields] OR “elderly”[All Fields] OR “elderlies”[All Fields]) AND ((“orexins”[MeSH Terms] OR “orexins”[All Fields] OR “orexin”[All Fields]) AND (“antagonist”[All Fields] OR “antagonists and inhibitors”[MeSH Subheading] OR “suvorexant”[All Fields] OR “lemborexant”[All Fields] OR “almorexant”[All Fields] OR “daridorexant”[All Fields] OR “orexin receptor antagonist”[All Fields]). The reference lists of retrieved papers and reviews were also screened for additional suitable sources.

### Eligibility criteria, study selection, and quality assessment

Inclusion criteria were: (1) randomized controlled trials (RCTs); (2) studies evaluating the use of DORAs; (3) CI in elderly patients (≥65 years). Exclusion criteria were: non-human studies, non-English-language studies, and different publication types (e.g. conference abstracts, reviews). All studies identified through the systematic search strategy in each database were imported into Rayyan (https://www.rayyan.ai/), where duplicate records were first removed. The selection process to determine study eligibility was then carried out in two distinct stages. In the initial phase, two independent reviewers (AA and ER) screened titles and abstracts against the predefined inclusion and exclusion criteria. In the second phase, the full texts of all articles considered potentially eligible were retrieved and independently reassessed by the same reviewers against the eligibility criteria. For all studies meeting the inclusion criteria, data extraction was then performed independently by both reviewers (AA and ER), who collected information on study characteristics and predefined outcome measures. In cases of disagreement about study inclusion or inconsistencies in data extraction, a third reviewer (FDL) was consulted to resolve discrepancies through discussion and consensus. Individual study quality was evaluated using Version 2 of the Cochrane risk-of-bias tool for randomized trials (RoB 2) [21].

### Data extraction

When outcomes were unavailable from a published article, supplementary materials, or a ClinicalTrials.gov registry entry, the corresponding author and/or the study sponsors were contacted three times over three weeks to request the necessary data. The extracted data included study characteristics such as the first author, publication year, and NCT identifier. Furthermore, we extracted data on total sample size, number of participants in the experimental and control groups, percentage of males, funding source, study design, active compound tested, dosage(s), type of control used, treatment duration, and time-points of assessment. Moreover, for both the experimental (i.e. DORAs) and control groups, we recorded efficacy outcomes, including the mean and the standard deviation (SD) for WASO, LPS, TST, SE, sTST, sWASO, and sSL. Additionally, we collected safety data (i.e. the number of participants assessed for safety and the number of TEAEs and SAEs), separately for both the experimental and control groups.

### Data synthesis and statistical analysis

Only phase III RCTs were included in the quantitative synthesis. For continuous outcomes, the results of single studies were combined using a random-effects model. In two of the included studies, the change from baseline in each efficacy outcome was expressed as least-square means [16, 22], instead of arithmetic means. Therefore, to keep different measures into the computation of the pooled efficacy estimates, we manually extracted, for each included study, the reported change score, and combined them adopting an inverse-variance method [23].

For the categorical outcomes, we computed summary risk ratios (RR) of the likelihood of detecting TEAEs and SAEs among treated versus untreated individuals. In all analyses, statistical heterogeneity was quantified using the I^2^ metric.

For each outcome, the total number of publications included in the meta-analyses was <10, thus we were not able to assess publication bias, either graphically, through funnel plots, or formally, through Egger’s regression asymmetry test (in such cases, the power is too low to distinguish chance from real asymmetry) [23]. All meta-analyses were performed using RevMan software, version 5.4 (Copenhagen: The Cochrane Collaboration, 2020).

## Results

### Study selection

**Figure 1:**
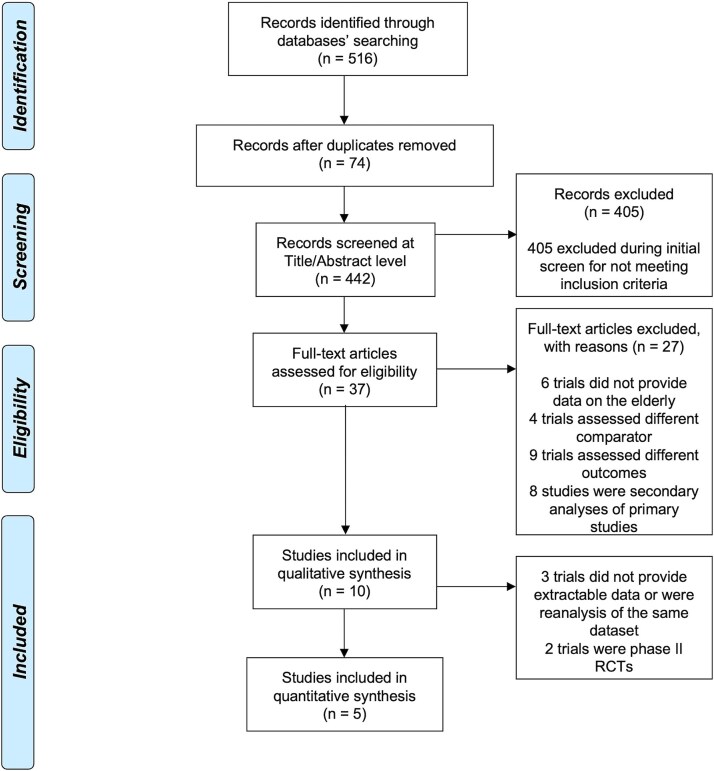
Prisma flow diagram.

The search strategy identified 516 reports. After duplicates removal, 442 records were screened based on title and abstract. Thus, in the subsequent phase, 37 articles were retrieved for full-text assessment. Finally, 10 items were included in the systematic review for qualitative synthesis. The other reports were excluded for the following reasons: 6 trials did not provide data on the elderly; 4 trials assessed a different comparator; 9 trials assessed different outcomes; 8 studies were secondary analyses of primary studies. We then selected trials for inclusion in the quantitative synthesis from the 10 remaining studies. Among these, 3 trials did not provide extractable data or were re-analyses of the same dataset [24–26], and 2 trials were phase 2 RCTs [27, 28]. Therefore, only 5 studies were included in the meta-analysis [16, 22, 29–31]. No relevant unpublished studies were identified following a search of ClinicalTrials.gov. Figure 1 provides an overview of the study selection procedure.

### Qualitative synthesis

Among the studies included in the systematic review, eight were phase 3 RCTs [16, 22, 24–26, 29–31], while two were phase 2 crossover RCTs [27, 28]. Of note, only two RCTs were specifically designed for elderly patients, while the other studies evaluated DORAs in adult CI patients. Therefore, in these cases, we reported data from a subgroup analysis of patients older than 65 years. The number of patients aged 65 years or older treated with DORAs ranged from 33 to 319 (for a total number of 2866), whereas those in the control group ranged from 9 to 318 (for a total number of 1780). The interventions were suvorexant in two studies [22, 25], almorexant in one study [27], lemborexant in three studies [26, 29, 31], and daridorexant in four studies [16, 24, 28, 30]. Different dosages of DORAs were employed across trials: suvorexant 15 mg (SUV15) and 30 mg (SUV30); almorexant 25 mg, 50 mg, 100 mg, and 200 mg; lemborexant 5 mg (LEM5) and 10 mg (LEM10); daridorexant 10 mg (DAR10), 25 mg (DAR25), and 50 mg (DAR50). In all included trials, DORAs were compared versus placebo, with the exception of one study [31] in which LEM5 and LEM10, administered in two separate study arms, were tested against Placebo and against Zolpidem. However, as no other RCT compared DORAs versus a different drug, we extracted only the data of the placebo arm as comparator. Treatment duration ranged from 15 days to six months, except for the two crossover studies, in which treatment with the investigational product lasted two days [27, 28]. In most studies, primary efficacy outcomes were assessed objectively using PSG (i.e. WASO, LPS, TST, SE), along with subjective sleep measures (sTST, sSL, sWASO, sSE). In some studies, efficacy outcomes were only based on subjective sleep measures [24, 25, 29]. All included studies analyzed the safety profile based on TEAEs, SAEs, or AEs that led to discontinuation. For a detailed overview of the included studies, see Table 1.

**Table 1:** Studies included in the qualitative synthesis

Author	Year	NCT	Study design	Total samples	Males %	Country	Funding	Active principle	Dose	Control type	Treatment duration	Time-points	Efficacy outcomes^*^	Safety outcomes	N. pts DORAs >65 years	N. pts control >65 years
Herring	2017	NCT01021813 (MK-4305-009 AM3)†	RCT phase III	779	44,0	Multicountry	Merck	SUV	30 mg	Placebo	3 months	m1; m3	sTST^*^; sTSO; sWASO	AEs	296	144
Herring (Study A)	2017	NCT01097629 (MK-4305-029) †	RCT phase III	839	35,4	Multicountry	Merck	SUV	15 mg; 30 mg	Placebo	3 months	m1; m3	WASO^*^; LPS^*^; sTST^*^; sSL; sWASO	SAEs	15 mg: 149; 30 mg: 88	144
Herring (Study B)	2017	NCT01097616 (MK-4305-028) †	RCT phase III	839	35,4	Multicountry	Merck	SUV	15 mg; 30 mg	Placebo	3 months	m1; m3	WASO^*^; LPS^*^; sTST^*^; sSL; sWASO	TEAEs; SAEs^*^	15 mg: 202; 30 mg: 319	318
Michelson	2014	NCT01021813	RCT phase III	781	45	Multicountry	Merck	SUV	30 mg	Placebo	12 months	m1; m12	sTST; sWASO; sSL	TEAEs; SAEs	308	151
Roth	2017	NCT00606593	RCT phase II	112	29,1	USA	Actelion Pharmaceuticals	ALM	25 mg; 50 mg; 100 mg; 200 mg	Placebo	Five 2-days treatments (cross-over study)	d2 (efficacy); d28 (safety)	WASO; TST; LPS; sWASO; sTST; sSL	TEAEs; SAEs	112	112
Murphy	2017	NCT01995838	RCT phase III	291	37,5	USA	Eisai	LEM	1 mg; 2.5 mg; 5 mg; 10 mg; 15 mg; 25 mg	Placebo	15 days	d1; d15	SE; LPS; WASO; sSE; sSL; sWASO	TEAEs; SAEs	33	9
Rosenberg	2019	NCT02783729	RCT phase III	1006	13,6	Multicountry	Eisai	LEM	5 mg; 10 mg	Placebo / Zolpidem	1 month	d2; m1	LPS^*^; WASO^*^; TST^*^; sSL, sWASO	TEAEs^*^; SAEs^*^	10 mg: 269; 5 mg: 255	208 (P); 263 (Z)
Zammit	2020	NCT02841709	RCT phase II	58	19	Germany and USA	Idorsia	DAR	5 mg; 10 mg; 25 mg; 50 mg	Placebo	Five 2-days treatments (cross-over study)	d2 (efficacy); d5 (safety)	WASO; TST; LPS; sWASO; sTST; sSL	TEAEs	56	54
Fietze	2022	NCT03545191	RCT phase III	930	33	Multicountry	Idorsia	DAR	25 mg; 50 mg	Placebo	3 months	m1; m3	WASO^*^; LPS^*^; sTST^*^	TEAEs^*^; SAEs^*^	50 mg: 121; 25 mg: 121	122
Mignot	2022	NCT03575104	RCT phase III	924	39	Multicountry	Idorsia	DAR	10 mg; 25 mg	Placebo	3 months	m1; m3	WASO^*^; LPS^*^; sTST^*^	TEAEs^*^; SAEs	10 mg: 121; 25 mg: 121	121
Kunz	2023	NCT03679884	RCT phase III	1684	NA	Multicountry	Idorsia	DAR	10 mg; 25 mg; 50 mg	Placebo	3 months	m3	sTST	TEAEs; SAEs	10 mg: 44; 25 mg: 39; 50 mg: 39	45
Arnold	2024	NCT02952820	RCT phase III	262	24	Multicountry	Eisai	LEM	5 mg; 10 mg	Placebo	6 months (PLAC); 6 months LEM5 vs LEM10	d7; m1; m2; m3; m4; m5; m6	sSL; sWASO; sTST; FSS for fatigue; ISI for insomnia severity	TEAEs^*^; SAEs^*^	5 mg: 87; 10 mg: 86	89

### Quality assessment

**Figure 2:**
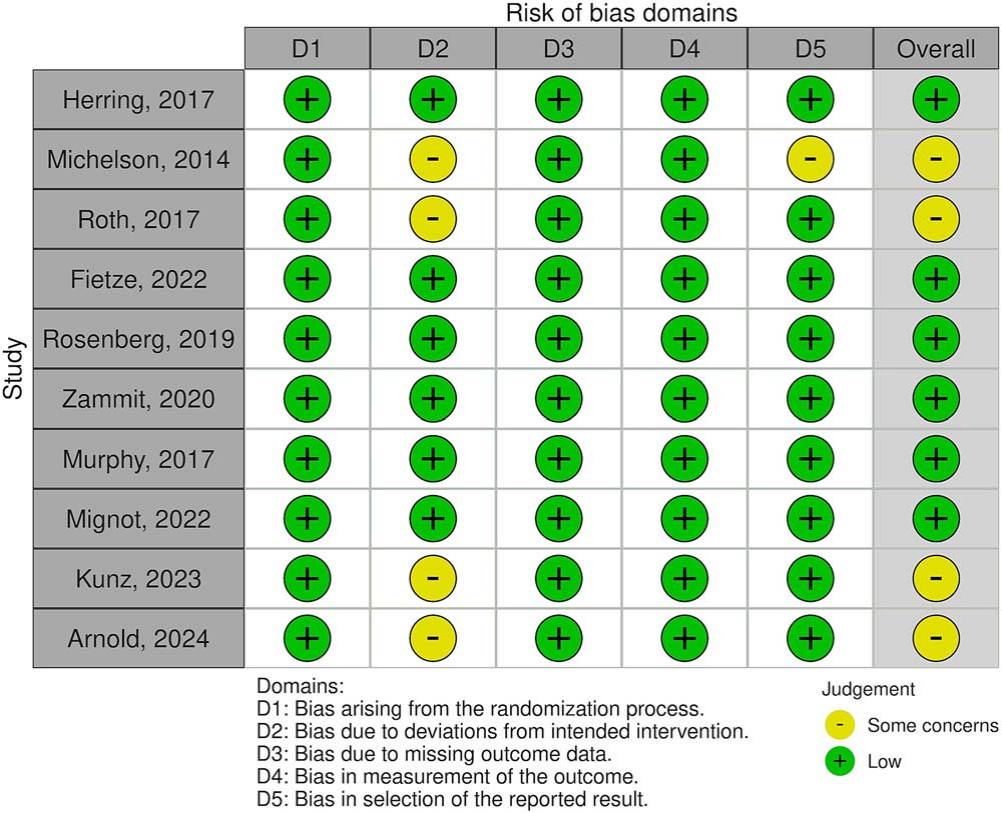
Quality assessment.

Quality assessment of each included study is detailed in Figure 2. According to the RoB 2 criteria, four studies raised some concerns about bias [24, 25, 27, 29], whereas the other studies were judged to have a low risk of bias. Specifically, two studies [24, 27] were judged to have some concerns about bias arising from deviations from the intended intervention (due to assignment to the intervention). Moreover, in three studies, some concerns about bias arose from adherence to the intervention [24, 25, 29]. Finally, the study by Michelson et al. raised concerns regarding the risk of bias in the selection of the reported results [25].

### Quantitative synthesis

Table 2 provides a detailed overview of the studies included in the quantitative synthesis.

**Table 2:** Studies included in the quantitative synthesis

Author	Year	NCT	Total samples	Males %	Country	Funding	Active principle	Dose	Control type	Treatment duration	Time-points	Efficacy outcomes^*^	Safety outcomes	N. pts DORAs >65 years	N. pts control >65 years
Herring	2017	NCT01021813 (MK-4305-009 AM3)†	779	44	Multicountry	Merck	SUV	30 mg	Placebo	3 months	m1; m3	sTST^*^; sSL; sWASO	TEAEs	296	144
Herring (Study A)	2017	NCT01097629 (MK-4305-029) †	839	35	Multicountry	Merck	SUV	15 mg; 30 mg	Placebo	3 months	m1; m3	WASO^*^; LPS^*^; sTST^*^; sSL; sWASO	SAEs	15 mg: 149; 30 mg: 88	144
Herring (Study B)	2017	NCT01097616 (MK-4305-028) †	839	35	Multicountry	Merck	SUV	15 mg; 30 mg	Placebo	3 months	m1; m3	WASO^*^; LPS^*^; sTST^*^; sSL; sWASO	TEAEs; SAEs^*^	15 mg: 202; 30 mg: 319	318
Rosenberg	2019	NCT02783729	1006	14	Multicountry	Eisai	LEM	5 mg; 10 mg	Placebo / Zolpidem	1 month	d2; m1	LPS^*^; WASO^*^; TST^*^; sSL, sWASO	TEAEs^*^; SAEs^*^	10 mg: 269; 5 mg: 255	208 (P); 263 (Z)
Fietze	2022	NCT03545191	930	33	Multicountry	Idorsia	DAR	25 mg; 50 mg	Placebo	3 months	m1; m3	WASO^*^; LPS^*^; sTST^*^	TEAEs^*^; SAEs^*^	50 mg: 121; 25 mg: 121	122
Mignot	2022	NCT03575104	924	39	Multicountry	Idorsia	DAR	10 mg; 25 mg	Placebo	3 months	m1; m3	WASO^*^; LPS^*^; sTST^*^	TEAEs^*^; SAEs	10 mg: 121; 25 mg: 121	121
Arnold	2024	NCT02952820	262	24	Multicountry	Eisai	LEM	5 mg; 10 mg	Placebo	6 months (PLAC); 6 months LEM5 vs LEM10	d7; m1; m2; m3; m4; m5; m6	sSL; sWASO; sTST; FSS for fatigue; ISI for insomnia severity	TEAEs^*^; SAEs^*^	5 mg: 87; 10 mg: 86	89

^*^Denotes the outcome(s) for which each publication provided enough data to be included in the pooled analyses of that specific outcome.

Abbreviations: ALM: almorexant; DAR: daridorexant; D: day; FSS: fatigue severity scale; ISI: insomnia severity index; LEM: lemborexant; M: month; PLAC/P: placebo; RCT: randomized controlled trial; SE: sleep efficiency; sTST: subjective total sleep time; SUV: suvorexant; Z: zolpidem. Studies identified as MK-4305-009 AM3, MK-4305-029 (Study A) and MK-4305-028 (Study B) are registered as separate trials on CT.gov, but their data have been reported into the same publication.

^†^Specific data on the elderly sub-sample were made available by the study sponsor, through access into its clinical trials registry platform.

#### Efficacy outcomes

A total of 2235 patients in the experimental group and 1409 patients in the control group were included in the quantitative synthesis. Most active treatments were associated with significant improvements in objective sleep measures at months 1 and 3 (Table 3).

**Table 3:** Results of the meta-analyses about efficacy outcomes at months 1 and 3

Outcomes	N. studies (sample)	N / N	Mean difference (95% CI)	p	I^2^, %
A. Month 1					
1. WASO					
– DAR10 [16]	1 (238)	121 / 117	0.01 (−10.2; 10.2)	0.9	—
– DAR25 [16, 30]	2 (360)	236 / 124	−13.0 (−13.8; −12.2)	<0.001	0
– DAR50 [30]	1 (243)	121 / 122	−21.2 (−22.0; −20.4)	<0.001	—
– LEM5 [31]§	1 (474)	266 / 208	−23.0 (−30.0: −16.1)	<0.001	—
– LEM10 [31]§	1 (477)	269 / 208	−23.5 (−30.5; −16.5)	<0.001	—
2. LPS					
– DAR25 [16, 30]	2 (475)	236 / 239	−8.6 (−10.1; −7.2)	<0.001	0
– DAR50 [30]	1 (243)	121 / 122	−13.7 (−14.3; −13.1)	<0.001	—
– LEM5 [31]§	1 (474)	266 / 208	−10.2 (−15.5; −5.0)	<0.001	—
– LEM10 [31]§	1 (477)	269 / 208	−13.2 (−18.0; −8.4)	<0.001	—
– SUV15 [22]^*^	2 (370)	135 / 235	−4.7 (−13.2; 3.7)	0.3	46
– SUV30 [22]^*^	2 (470)	235 / 235	−7.0 (−14.6; 0.5)	0.07	38
3. TST					
– LEM5 [31]§	1 (474)	266 / 208	34.1 (33.6; 34.6)	<0.001	—
– LEM10 [31]§	1 (477)	269 / 208	38.8 (38.3; 39.3)	<0.001	—
4. sTST					
– DAR10 [16]	1 (238)	121 / 117	12.3 (−1.14; 25.7)	0.07	—
– DAR25 [16, 30]	2 (475)	236 / 239	20.5 (10.7; 30.3)	<0.001	99
– SUV15 [22]^*^	2 (488)	191 / 297	16.3 (4.4; 28.2)	0.007	0
– SUV30 [22]^*^	3 (1040)	600 / 440	22.5 (4.2; 30.8)	<0.001	0
B. Month 3					
1. WASO					
– DAR10 [16]	1 (219)	110 / 109	0.01 (−10.6; 10.6)	0.9	—
– DAR25 [16, 30]	2 (460)	229 / 231	−16.2 (−18.0; −14.3)	<0.001	74
– DAR50 [30]	1 (243)	121 / 122	−19.6 (−20.5; −18.7)	<0.001	—
2. LPS					
– DAR25 [16, 30]	2 (460)	229 / 231	−6.8 (−9.0; −4.5)	<0.001	88
– DAR50 [30]	1 (243)	121 / 122	−14.9 (−15.6; −14.2)	<0.001	—
– SUV15 [22]^*^	2 (349)	131 / 218	−6.8 (−18.5; 4.9)	0.3	52
– SUV30 [22]^*^	2 (437)	219 / 218	−9.4 (−18.7; −0.01)	0.049	38
3. sTST					
– DAR10 [16]	1 (219)	110 / 109	12.5 (−0.54; 25.5)	0.06	—
– DAR25 [16, 30]	2 (465)	229 / 236	23.1 (14.0; 32.2)	<0.001	96
– SUV15 [22]^*^	2 (458)	182 / 276	17.9 (6.1; 29.8)	0.003	0
– SUV30 [22]^*^	3 (917)	530 / 387	19.3 (10.6; 28.1)	<0.001	0

Objective (wake after sleep onset—WASO, latency to persistent sleep—LPS, and total sleep time—TST) and subjective (subjective total sleep time—sTST) efficacy outcomes, at month 1 and month 3, among elderly subjects treated with different DORAs, versus controls. Abbreviations: CI: confidence Interval; DAR10: daridorexant 10 mg/day; DAR 25: daridorexant 25 mg/day; DAR50: daridorexant 50 mg/day; LEM5: lemborexant 5 mg/day; LEM10: lemborexant 10 mg/day; N / N: total number of subjects in the experimental (DORAs) and control (placebo) group, respectively; SUV15: suvorexant 15 mg/day; SUV30: suvorexant 30 mg/day. In all analyses, the placebo was the control treatment.

^*^The analyses on the efficacy of SUV 15 and SUV 30 were based upon raw data reported across different datasets, providing specific estimates for the elderly: although registered as separate trials on CT.gov, the final results of these studies were combined and published into a single publication.

^§^The efficacy of LEM5 and LEM10 is assessed only against placebo.

**Figure 3:**
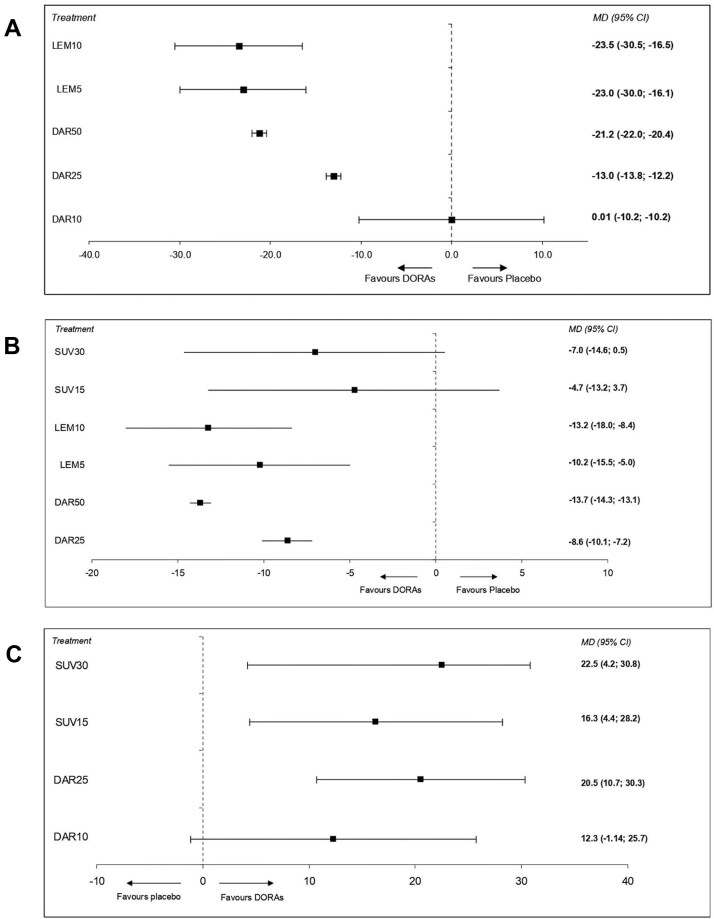
Results of quantitative synthesis regarding efficacy outcomes at month 1. (A) WASO at month 1; (B) LPS at month 1; (C) sTST at month 1. CI: confidence interval; DAR10: daridorexant 10 mg/day; DAR 25: daridorexant 25 mg/day; DAR50: daridorexant 50 mg/day; LEM5: lemborexant 5 mg/day; LEM10: lemborexant 10 mg/day; MD: mean difference; SUV15: suvorexant 15 mg/day; SUV30: suvorexant 30 mg/day.

Specifically, at month 1: compared to placebo, DAR25 reduced WASO by -13.0 minutes (95% CI, -13.8; -12.2), and DAR50 produced a greater reduction of -21.2 minutes (95% CI, -22.0; -20.4), while DAR10 did not lead to a statistically significant reduction. Similarly, LEM5 reduced WASO by -23.0 minutes (95% CI, -30.0; -16.1) and LEM10 by -23.5 minutes (95% CI, -30.5; -16.5) (Figure 3A). In addition, DAR25 reduced LPS by -8.6 minutes (95% CI, -10.1; -7.2), while DAR50 by -13.7 minutes (95% CI, -14.3; -13.1); LEM5 reduced LPS by -10.2 minutes (95% CI, -15.5; -5.0), and LEM10 by -13.2 minutes (95% CI, -18.0; -8.4); conversely, neither SUV15 nor SUV30 lead to a statistically significant reduction of LPS (Figure 3B). Finally, LEM5 increased TST by 34.1 minutes (95% CI, 33.6; 34.6), and LEM10 by 38.8 minutes (95% CI, 38.3; 39.3). See also supplementary materials (Figures S1, S2, S7, and  S8).

**Figure 4:**
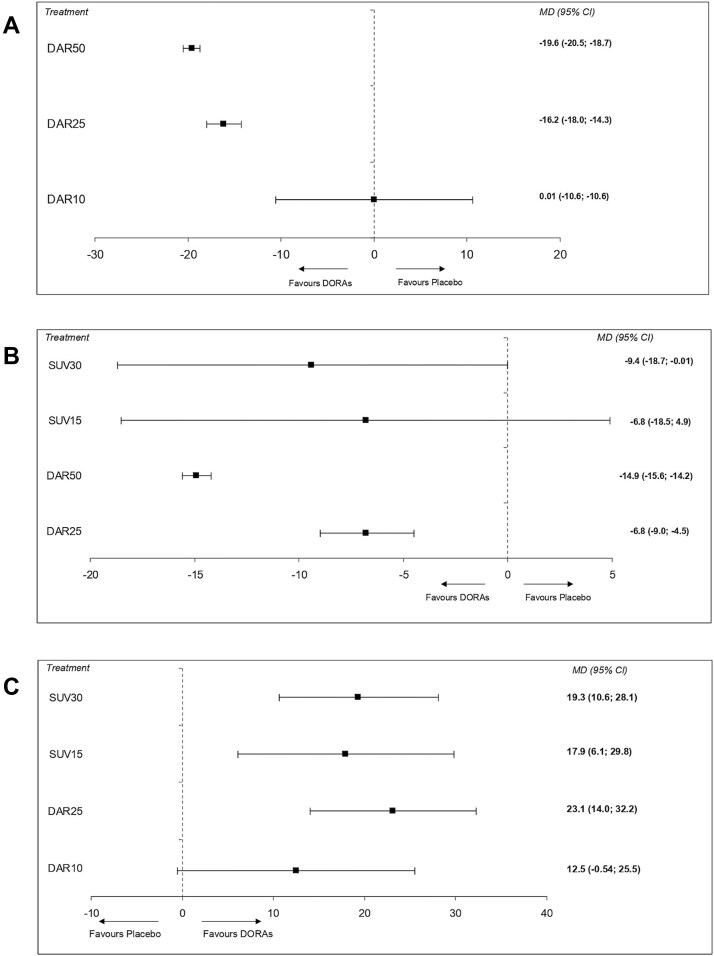
Results of quantitative synthesis regarding efficacy outcomes at month 3. (A) WASO at month 3; (B) LPS at month 3; (C) sTST at month 3. CI: confidence interval; DAR10: daridorexant 10 mg/day; DAR 25: daridorexant 25 mg/day; DAR50: daridorexant 50 mg/day; MD: mean difference; SUV15: suvorexant 15 mg/day; SUV30: suvorexant 30 mg/day.

At month 3, compared to placebo: DAR25 reduced WASO by -16.2 minutes (95% CI, -18.0; -14.3) and DAR50 by -19.6 minutes (95% CI, -20.5; -18.7) (Figure 4A); DAR25 reduced LPS by -6.8 minutes (95% CI, -9.0; -4.5) and DAR50 by -14.9 minutes (95% CI, -15.6; -14.2). SUV30 significantly improved LPS by -9.4 minutes (95% CI -18.7; -0.01), while SUV15 did not lead to a significant reduction in LPS (Figure 4B). See also supplementary materials (Figures S4, S5, S11, and  S12).

Regarding subjective sleep measures, DAR10 did not improve sTST at either month 1 or month 3, whereas DAR25 increased sTST at month 1 by 20.5 minutes (95% CI, 10.7; 30.3) and at month 3 by 23.1 minutes (95% CI, 14.0; 32.2). Finally, both dosages of SUV improved sTST at both month 1 (SUV 15 by 16.3 minutes, 95% CI 4.4; 28.2; SUV 30 by 22.5 minutes, 95% CI 4.2; 30.8) and month 3 (SUV15 by 17.9 minutes, 95% CI 6.1; 29.8; SUV30 by 19.3 minutes, 95% CI 10.6; 28.1) (Figures 3C and 4C). See also supplementary materials (Figures S3, S6, S9, S10, S13, and  S14).

#### Safety outcomes

DAR10 did not increase the risk of TEAEs compared with placebo, nor did DAR25 or DAR50 (Table 4 and Figure S15). Moreover, DAR25 and DAR50 were not associated with increased risk of SAEs. In contrast, LEM5 (52/352 vs. 29/298; RR 1.60, 95% CI 1.05; 2.44, Figure S16) and LEM10 (67/352 vs. 29/298; RR 2.07, 95% CI 1.39; 3.10, Figure S18) increased the risk of TEAEs, but not the risk of SAEs (Figures S17 and S19). SUV15 was not associated with increased risk of TEAEs and SAEs, while SUV30 increased the risk of TEAEs (149/627 vs. 69/469; RR 1.62, 95% CI 1.25; 2.09). A detailed overview of safety is depicted in Table 4 and Table S1.

**Table 4:** Results of the meta-analyses about safety outcomes

Outcomes	N. studies (sample)	Total subjects (n/N vs. n/N)	RR (95% CI)	p	I^2^,%
DAR10					
– TEAEs [16]	1 (241)	52/121 vs. 47/120	1.10 (0.81–1.49)	0.6	—
DAR25					
– TEAEs [16, 30]	2 (484)	94/242 vs. 85/242	1.11 (0.88–1.40)	0.4	0
– SAEs [30]	1 (243)	1/121 vs. 3/122	0.34 (0.04–3.19)	0.3	—
DAR50					
– TEAEs [30]	1 (241)	42/119 vs. 38/122	1.13 (0.79–1.62)	0.5	—
– SAEs [30]	1 (241)	0/119 vs. 3/122	0.15 (0.01–2.80)	0.2	—
LEM5					
– TEAEs [29, 31]§	2 (650)	52/352 vs. 29/298	1.60 (1.05–2.44)	0.03	0
– SAEs [29, 31]§	2 (650)	5/352 vs. 1/298	3.38 (0.56–20.5)	0.2	0
LEM10					
– TEAEs [29, 31]§	2 (650)	67/352 vs. 29/298	2.07 (1.39–3.10)	<0.001	0
– SAEs [29, 31]§	2 (650)	3/352 vs. 1/298	1.82 (0.32–10.4)	0.5	0
SUV15					
– TEAEs [22]	1 (671)	42/202 vs. 69/469	1.41 (1.00–2.00)	0.051	—
– SAEs [22]	1 (671)	3/202 vs. 11/469	0.63 (0.18–2.25)	0.5	—
SUV30					
– TEAEs [22]	1 (1096)	149/627 vs. 69/469	1.62 (1.25–2.09)	0.003	—
– SAEs [22]	1 (1096)	12/627 vs. 11/469	0.82 (0.36–1.83)	0.6	—

Results of the head-to-head meta-analyses comparing the risk of TEAEs and of SAEs among elderly subjects treated with different DORAs, versus untreated subjects.

Abbreviations: CI: confidence interval; DAR 10: daridorexant 10 mg/day; DAR 25: daridorexant 25 mg/day; LEM5: lemborexant 5 mg/day; LEM10: lemborexant 10 mg/day; n/N: number of subjects reporting ≥1 AE / total number of subjects in the experimental (DORAs) and control (placebo) group, respectively; RR: risk ratio; SUV15: suvorexant 15 mg/day; SUV30: suvorexant 30 mg/day. In all analyses, placebo was the control. A TEAE was defined as an AE emerging on or after the first administration of a study drug, up to 14 days after the last dose.

^§^The safety of LEM5 and LEM10 is assessed only against placebo.

## Discussion

In this systematic review and meta-analysis, we examined the efficacy and safety of DORAs in a specific and relevant patient population, namely older adults. The key finding of the present meta-analysis is that DORAs demonstrate overall good efficacy and safety compared with placebo in this population, confirming their role as a valuable therapeutic option for the elderly. In particular, we were able to summarize evidence on daridorexant’s improvements in objective and subjective sleep measures at both 1 and 3 months of treatment, whereas suvorexant demonstrated improvements mostly after 3 months of sustained treatment. Regarding lemborexant, evidence of its efficacy is limited to a 1-month treatment period. Importantly, all molecules were well tolerated in this vulnerable population.

A previous network meta-analysis demonstrated that DORAs are the most suitable recommendation for CI because of their more favorable efficacy and safety profile compared with other insomnia treatments [32]. However, this study did not specifically assess the effects of DORAs in older adults, underscoring the need for further research on the benefit-to-risk ratio of these agents in this patient subgroup. Here, we sought to address this gap by specifically assessing DORAs in the elderly. In line with this, we performed a systematic literature search of all studies comparing DORAs with placebo or other hypnotics.

We identified a small number of studies that included this specific target population. In all cases but one, the comparator was placebo. Only the study by Rosenberg et al. included a zolpidem treatment arm, reporting significantly greater improvements in objective sleep parameters with lemborexant than with zolpidem [31]. However, having only one study that used zolpidem as a comparison limits our ability to summarize the findings. Notably, although this review focuses on elderly people, the available evidence largely derives from RCTs conducted in the general adult population, with older adults represented primarily through predefined subgroups or secondary analyses. Indeed, only two RCTs [22, 29] specifically investigated DORAs in people over 65 years, whereas most RCTs were conducted in the general population, including only a small proportion of elderly patients. Accordingly, the present work constitutes a systematic review and meta-analysis of randomized evidence for elderly subgroups rather than elderly-only RCTs. Several eligible trials could not be included in the quantitative synthesis because the subgroup data were not extractable. These studies were therefore summarized qualitatively, revealing substantial heterogeneity in treatment duration (ranging from 2 days to 6 months), dosages, and sample size. Of note, efficacy outcomes were similar across studies, though heterogeneity was observed in the inclusion of both subjective and objective efficacy measures. Conversely, quality assessment revealed that the included studies were of high overall quality, with only four studies raising some concerns about risk of bias, thereby strengthening the reliability of our findings.

It is worth noting that the results of our quantitative synthesis are limited to agents for which outcome data for the specific target population were provided by the Authors. In particular, the efficacy outcomes data are available only for daridorexant, suvorexant, and lemborexant. This prevented us from evaluating these specific points for almorexant. All treatments showed a dose–response effect on efficacy endpoints (namely WASO and LPS) at month 1, except for SUV15 (concerning LPS) and DAR10. However, it is worth mentioning that DAR10 did not meet the primary endpoints in the general population cohort as well [16]. Our study could not provide valuable information on TST and SE for our population of interest, due to a lack of available data. In particular, TST was analyzed solely in the phase II RCTs, which were excluded from our quantitative synthesis due to our prespecified inclusion criteria and the study design (i.e. crossover studies with only two days of treatment duration), while SE was analyzed only in the study by Murphy et al., for which data were not extractable for the elderly [26–28]. Nevertheless, we report reliable data on the efficacy of DORAs in improving subjective measures, such as sTST, consistently at months 1 and 3, supporting the clinical significance of our findings and their relevance from a patient perspective.

A critical focus of our study was the analysis of the safety profile. Regarding lemborexant, both doses increased the incidence of TEAEs compared with placebo. In both studies, the most frequent AEs were somnolence and headache [29, 31]. Consistently, in a network meta-analysis comparing lemborexant, suvorexant, and daridorexant in the general population, lemborexant was associated with the highest risk of somnolence and treatment discontinuation [15]. Nevertheless, importantly, neither dose of lemborexant increased the risk of SAEs in our population of interest. Conversely, daridorexant across all explored doses demonstrated a favorable tolerability profile, with no increase in the risk of TEAEs or SAEs in this vulnerable population. Our analysis focused on a limited 3-month treatment duration. However, long-term data on DAR50 in a large cohort of adult patients with CI demonstrate its sustained efficacy with no safety concerns [24]. The most frequently reported TEAEs were headache, somnolence, fatigue, and dizziness, with rates comparable to those in the placebo group (few cases, approximately 5% or less). Notably, a limited number of falls were reported with both DAR25 and DAR50, even though the number of cases was lower than in the placebo group. Accordingly, a recent meta-analysis demonstrated that DORAs did not increase the risk of falls or fractures, even at high doses (i.e. DAR50 or SUV30) [33]. Indeed, similar AEs were reported in the suvorexant study, namely headache, somnolence, dizziness, and fatigue [22]. In particular, the incidence of somnolence and fatigue increased with dose. However, for this drug as well, SAEs were not significantly higher than with placebo. Moreover, the treatment was associated with a low discontinuation rate due to AEs, which did not differ from that observed with placebo. Of note, CI in older adults frequently co-occurs with obstructive sleep apnea (OSA; Comorbid Insomnia with Sleep Apnea, COMISA) [34–36]. Although COMISA was not a primary focus of the present study, post-hoc analyses indicate that DAR50 is effective compared with placebo in patients with comorbid insomnia and mild OSA, including individuals older than 65 years [37]. Although no age-specific subgroup analyses were performed, the available data suggest that the safety profile of daridorexant is comparable between patients with and without OSA [37], supporting its possible use also in older COMISA patients, even though the available evidence is still limited.

Overall, data from our quantitative synthesis, along with growing evidence from the general population, indicate the safety profile of DORAs for CI. Consistently, in a recently published network meta-analysis comparing pharmacological treatments for CI, lemborexant and eszopiclone had the most favorable profiles for efficacy and acceptability, although eszopiclone was associated with a significant risk of AEs [38]. The subgroup analysis of the elderly population revealed a significantly increased risk of any AEs in patients taking short-, intermediate-, and long-acting BDZ, confirming the well-known concerns on use of this class of agents in this specific population [38]. Accordingly, a previous network meta-analysis comparing hypnotics in older adults demonstrated a substantially increased risk of AEs during treatment with triazolam [39]. However, neither of these previous meta-analyses specifically addressed DORAs in the elderly. Moreover, the studies included in our meta-analysis only employed placebo as comparator. Therefore, we cannot draw conclusions about the safety profile of DORAs in comparison with other hypnotic drugs in this subgroup of patients.

Treating CI in older adults is particularly challenging, as clinicians must balance efficacy against the risk of AEs in a fragile, polymorbid population often exposed to polypharmacy [6]. Traditional hypnotics, including BDZ and BZRA, have been associated with increased stroke risk and cognitive decline [40, 41]. At the same time, sleep disruption may contribute to neurodegeneration by impairing glymphatic clearance, which is most active during slow-wave sleep and is implicated in amyloid-β accumulation [42, 43]. In this context, preclinical evidence suggests that DORAs may not only improve sleep but also restore sleep–wake regulation, enhance glymphatic function, and potentially exert neuroprotective effects, including reductions in amyloid-β burden and tau-mediated neurodegeneration [44, 45]. Therefore, even if the current evidence remains limited, DORAs may also indirectly exert an influence within the neurophysiological cascade of neurodegenerative disorders, particularly Alzheimer’s disease. Future longitudinal and mechanistic studies are needed to determine whether improvements in sleep continuity mediated by DORAs translate into meaningful cognitive or neurodegenerative outcomes in older adults.

We conducted a comprehensive systematic review of the evidence on the use of DORAs in the elderly. The main strength of the present study is the novelty of the topic, which, to the best of our knowledge, has not been addressed yet. Moreover, the aim of the present review is clinically meaningful, providing support for the efficacy and tolerability profile of DORAs in older adults, a complex subgroup of patients with limited safe therapeutic options. However, our study acknowledges several limitations. First, the randomized evidence currently available for the elderly subjects is scarce, almost entirely based upon few studies directly comparing DORAs versus placebo. This, inevitably, limits the generalizability of the present findings and does not allow to infer any clinically meaningful conclusion on the role of DORAs compared to other hypnotic drugs in this age group, nor to compare drug classes among themselves, such as in a network comparative meta-analysis or dose–response model-based network meta-analysis. Given the limited availability of RCTs exclusively enrolling older adults with CI, this review included studies that reported efficacy and safety data for participants aged 65 years or older, either as primary analyses or as predefined subgroup or secondary analyses within larger adult trials. Furthermore, we were able to extract data from only a small proportion of studies due to a lack of data publication and data-sharing restrictions. Moreover, quantitative synthesis was further limited by heterogeneity across studies in the timepoints and outcome measures used. For these reasons, we could perform a quantitative synthesis for only a few outcomes and/or interventions. Considering the relevance of the topic, future, real-world studies and, ideally, high-quality research efforts comparing the safety and efficacy of DORAs with traditional hypnotics are needed to inform clinical practice. Finally, we could provide only quantitative information for a limited timeframe (i.e. the follow-up period for the studies was up to 3 months).

## Conclusion

Although the available data on the topic are limited, this quantitative synthesis provides the most comprehensive, up-to-date evidence on the use of DORAs in older adults, supporting their use as an effective and safe therapeutic option for the treatment of CI in this complex patient population. Nonetheless, the limitations of this study underscore the need for future RCTs specifically addressing our target population, thereby expanding knowledge of the use of these drugs. Moreover, future real-world studies focused on the use of DORAs in the elderly are needed to assess their effectiveness and safety profiles in clinical practice and to provide information on their long-term use. Finally, their cost-effectiveness ratio should be analysed in this population.

## Supplementary material

Supplementary material is available at *SLEEP* online.

## Supplementary Material

Supplementary_material_zsag135

## Data Availability

All data used for the analyses are available in the original articles cited, in their supplementary materials, or on ClinicalTrials.gov. In some instances, the raw data were acquired via an agreement with Merck & Co.©, which should be contacted for additional details. Further enquiries can be directed to the corresponding authors.
