# Supplementary material for: Treatment of chronic insomnia in elderly adults with dual orexin receptor antagonists: a systematic review and meta-analysis
Source: Sleep. 2026 May 17;49(8):zsag135. doi: 10.1093/sleep/zsag135 (PMC13472891; doi:10.1093/sleep/zsag135)
Supplement: Supplementary file 1 [file supplementary_material_zsag135.zip › Supplementary_Material_Accepted_zsag135.docx]

**Treatment of chronic insomnia in elderly adults with dual orexin receptor antagonists: A systematic review and meta-analysis**

Eleonora Rollo^1,2,*^, Annibale Antonioni^3,4,*^, Lamberto Manzoli^5,6^, Francesco Di Lorenzo^7, †^, Maria Elena Flacco^6, †^

^1^Center for Neurodegenerative Diseases and the Aging Brain, University of Bari Aldo Moro at Pia Fondazione "Card. G. Panico", Tricase, Italy

^2^Department of Neurosciences, Università Cattolica del Sacro Cuore, Rome, Italy.

^3^Department of Neuroscience and Rehabilitation, University of Ferrara, 44121 Ferrara, Italy.

^4^IIT@UniFe Center for Translational Neurophysiology of Speech and Communication, Istituto Italiano di Tecnologia, 44121 Ferrara, Italy

^5^Department of Medical and Surgical Sciences, University of Bologna, 40100 Bologna, Italy.

^6^Department of Environmental and Prevention Sciences, University of Ferrara, 44121 Ferrara, Italy

^7^Neuropsychophysiology Lab, Santa Lucia Foundation IRCCS, 00179, Rome, Italy.

*The two first Authors have contributed equally to manuscript and share co-first authorship

^†^The two last Authors have contributed equally to the manuscript and share co-last Authorship

**Corresponding Author:**

Dr. Annibale Antonioni

Department of Neuroscience and Rehabilitation, University of Ferrara, Via Ludovico Ariosto 35, 44121 Ferrara, Italy.

[annibale.antonioni@edu.unife.it](mailto:annibale.antonioni@edu.unife.it)

**Table S1:** Absolute number of treatment-emergent adverse events (TEAEs) reported in each study, stratified by treatment dose.

| **DORA** | **Herring et al. 2017** | **Rosenberg et al. 2019** | **Fietze et al. 2022** | **Mignot et al. 2022** | **Arnold et al. 2024** |
| --- | --- | --- | --- | --- | --- |
| **SUV15** | Somnolence (5.4)  Headache (6.9)  Fatigue (1.5)  Dry mouth (2.5)  Nasopharyngitis (3.5)  Upper respiratory tract infection (3.0)  Dizziness (4.0)  Diarrhea (4.0)  Abnormal dreams (2.5)  Urinary tract infection (3.0)  Back pain (2.0)  Cough (2.5)  Nausea (2.0)  Pruritis (2.0)  Gastroenteritis (2.0) |  |  |  |  |
| **SUV30** | Somnolence (8.8)  Headache (5.6)  Fatigue (3.8)  Dry mouth (2.9)  Nasopharyngitis (2.9)  Upper respiratory tract infection (2.6)  Dizziness (2.2)  Diarrhea (2.2)  Abnormal dreams (1.6)  Urinary tract infection (1.3)  Drug administration error (1.3)  Back pain (1.1)  Cough (1.1)  Nausea (1.1)  Pruritus (0.3)  Gastroenteritis (0.3) |  |  |  |  |
| **LEM5** |  | Headache (6.4)  Somnolence (4.1)  Urinary tract infection (1.1)  Nasopharyngitis (2.6)  Upper respiratory tract infection (2.3)  Dizziness (1.1) |  |  | Somnolence (8.1)  Nasopharyngitis (5.8)  Headache (9.3) |
| **LEM10** |  | Headache (4.9)  Somnolence (7.1)  Urinary tract infection (3.4)  Nasopharyngitis (0.4)  Upper respiratory tract infection (0.4)  Dizziness (0.7) |  |  | Somnolence (19.0)  Nasopharyngitis (7.1)  Headache (1.2) |
| **DAR10** |  |  |  | Headache (4.1)  Nasopharyngitis (12.4)  Somnolence (2.5)  Fatigue (3.3)  Dizziness (1.7)  Diarrhoea (2.5)  Fall (1.7)  Upper respiratory tract infection (2.5)  Upper abdominal pain (2.5)  Hypertension (0.8)  Cough (1.7)  Blood creatine kinase increased (2.5) |  |
| **DAR25** |  |  | Nasopharyngitis (1.7)  Headache (4.1)  Fatigue (3.3)  Accidental overdose (0.8)  Dizziness (3.3)  Back pain (1.7)  Somnolence (5.0)  Fall (0.8)  Upper abdominal pain (2.5) | Headache (5.8)  Nasopharyngitis (5.0)  Somnolence (5.0)  Fatigue (5.8)  Dizziness (2.5)  Fall (2.5)  Upper respiratory tract infection (0.8)  Cough (2.5)  Blood creatine kinase increased (0.8) |  |
| **DAR50** |  |  | Nasopharyngitis (5.9)  Headache (5.0)  Nausea (3.4)  Fatigue (2.5)  Accidental overdose (2.5)  Dizziness (0.8)  Back pain (0.8)  Somnolence (0.8)  Diarrhoea (0.8)  Fall (0.8) |  |  |
| **PLAC** | Somnolence (3.2)  Headache (5.3)  Fatigue (1.7)  Dry mouth (1.7)  Nasopharyngitis (4.1)  Upper respiratory tract infection (1.3)  Dizziness (4.9)  Diarrhea (1.5)  Abnormal dreams (0.9)  Urinary tract infection (2.3)  Drug administration error (2.1)  Back pain (2.8)  Cough (1.1)  Nausea (1.3)  Pruritus (0.9)  Gastroenteritis (0.6) | Headache (6.2) Somnolence (1.9) Urinary tract infection (1.0) Nasopharyngitis (1.4) Upper respiratory tract infection (1.9) Dizziness (1.9) | Nasopharyngitis (3.3)  Headache (4.1)  Nausea (0.8)  Fatigue (0.8)  Dizziness (0.8)  Back pain (0.8)  Somnolence (0.8)  Diarrhoea (0.8)  Fall (3.3)  Influenza (1.6)  Upper abdominal pain (0.8) | Headache (5.8) Nasopharyngitis (5.8) Somnolence (1.7) Fatigue (0.8) Dizziness (3.3) Diarrhoea (0.8) Fall (1.7) Upper respiratory tract infection (3.3) Upper abdominal pain (0.8) Hypertension (2.5) | Nasopharyngitis (9.0)  Headache (6.7) |

Numbers reported in brackets are the percentage of patients experiencing TEAEs in the elderly subgroup for each treatment arm.

**Figure S1**. Results of the meta-analysis comparing the mean wake after sleep onset (WASO) between subjects treated with Daridorexant - 25mg/day (DAR25) and untreated subjects - One-month follow-up data.

**Figure S2**. Results of the meta-analysis comparing the mean latency to persistent sleep (LPS) between subjects treated with Daridorexant - 25mg/day (DAR25) and untreated subjects - One-month follow-up data.

**Figure S3**. Results of the meta-analysis comparing the mean subjective total sleep time (sTST) between subjects treated with Daridorexant - 25mg/day (DAR25) and untreated subjects - One-month follow-up data.

**Figure S4**. Results of the meta-analysis comparing the mean wake after sleep onset (WASO) between subjects treated with Daridorexant - 25mg/day (DAR25) and untreated subjects - Three-month follow-up data.

**Figure S5**. Results of the meta-analysis comparing the mean latency to persistent sleep (LPS) between subjects treated with Daridorexant - 25mg/day (DAR25) and untreated subjects - Three-month follow-up data.

**Figure S6**. Results of the meta-analysis comparing the mean subjective total sleep time (sTST) between subjects treated with Daridorexant - 25mg/day (DAR25) and untreated subjects - Three-month follow-up data.

**Figure S7**. Results of the meta-analysis comparing the mean latency to persistent sleep (LPS) between subjects treated with Suvorexant - 15mg/day (SUV15) and untreated subjects - One-month follow-up data.

**Figure S8**. Results of the meta-analysis comparing the mean latency to persistent sleep (LPS) between subjects treated with Suvorexant - 30mg/day (SUV30) and untreated subjects - One-month follow-up data.

**Figure S9**. Results of the meta-analysis comparing the mean subjective total sleep time (sTST) between subjects treated with Suvorexant - 15mg/day (SUV15) and untreated subjects - One-month follow-up data.

**Figure S10**. Results of the meta-analysis comparing the mean subjective total sleep time (sTST) between subjects treated with Suvorexant - 30mg/day (SUV30) and untreated subjects - One-month follow-up data.

**Figure S11**. Results of the meta-analysis comparing the mean latency to persistent sleep (LPS) between subjects treated with Suvorexant - 15mg/day (SUV15) and untreated subjects - Three-month follow-up data.

**Figure S12**. Results of the meta-analysis comparing the mean latency to persistent sleep (LPS) between subjects treated with Suvorexant - 30mg/day (SUV30) and untreated subjects - Three-month follow-up data.

**Figure S13**. Results of the meta-analysis comparing the mean subjective total sleep time (sTST) between subjects treated with Suvorexant - 15mg/day (SUV15) and untreated subjects - Three-month follow-up data.

**Figure S14**. Results of the meta-analysis comparing the mean subjective total sleep time (sTST) between subjects treated with Suvorexant - 30mg/day (SUV30) and untreated subjects - Three-month follow-up data.

**Figure S15**. Results of the meta-analysis comparing the likelihood of treatment-emergent adverse events (TEAEs) among subjects treated with Daridorexant - 25mg/day (DAR25) versus untreated subjects.

**Figure S16**. Results of the meta-analysis comparing the likelihood of treatment-emergent adverse events (TEAEs) among subjects treated with Lemborexant - 5mg/day (LEM5) versus untreated subjects.

**Figure S17**. Results of the meta-analysis comparing the likelihood of serious adverse events (SAEs) among subjects treated with Lemborexant - 5mg/day (LEM5) versus untreated subjects.

**Figure S18**. Results of the meta-analysis comparing the likelihood of treatment-emergent adverse events (TEAEs) among subjects treated with Lemborexant - 10mg/day (LEM10) versus untreated subjects.

**Figure S19**. Results of the meta-analysis comparing the likelihood of serious adverse events (SAEs) among subjects treated with Lemborexant - 10mg/day (LEM10) versus untreated subjects.
